# Oncogenic RAS Mutants Confer Resistance of RMS13 Rhabdomyosarcoma Cells to Oxidative Stress-Induced Ferroptotic Cell Death

**DOI:** 10.3389/fonc.2015.00131

**Published:** 2015-06-22

**Authors:** Christina Schott, Ulrike Graab, Nicole Cuvelier, Heidi Hahn, Simone Fulda

**Affiliations:** ^1^Institute for Experimental Cancer Research in Pediatrics, Goethe-University, Frankfurt, Germany; ^2^German Cancer Consortium (DKTK), Heidelberg, Germany; ^3^German Cancer Research Center (DKFZ), Heidelberg, Germany; ^4^Institute of Human Genetics, University Medical Center, Göttingen, Germany

**Keywords:** rhabdomyosarcoma, RAS, cell death, apoptosis, ROS

## Abstract

Recent genomic studies revealed a high rate of recurrent mutations in the RAS pathway in primary rhabdomyosarcoma (RMS) samples. In the present study, we therefore investigated how oncogenic RAS mutants impinge on the regulation of cell death of RMS13 cells. Here, we report that ectopic expression of NRAS12V, KRAS12V, or HRAS12V protects RMS13 cells from oxidative stress-induced cell death. RMS13 cells engineered to express NRAS12V, KRAS12V, or HRAS12V were significantly less susceptible to loss of cell viability upon treatment with several oxidative stress inducers including the thioredoxin reductase inhibitor Auranofin, the glutathione (GSH) peroxidase 4 inhibitor RSL3 or Erastin, an inhibitor of the cysteine/glutamate amino acid transporter system ${x_{c}}^{-}$ that blocks GSH synthesis. Notably, addition of Ferrostatin-1 confers protection against Erastin- or RSL3-induced cytotoxicity, indicating that these compounds trigger ferroptosis, an iron-dependent form of programed cell death. Furthermore, RMS13 cells overexpressing oncogenic RAS mutants are significantly protected against the dual PI3K/mTOR inhibitor PI103, whereas they are similarly sensitive to DNA-damaging drugs such as Doxorubicin or Etoposide. This suggests that oncogenic RAS selectively modulates cell death pathways triggered by cytotoxic stimuli in RMS13 cells. In conclusion, our discovery of an increased resistance to oxidative stress imposed by oncogenic RAS mutants in RMS13 cells has important implications for the development of targeted therapies for RMS.

## Introduction

Rhabdomyosarcoma is the most common soft-tissue sarcoma in childhood and adolescence and can be divided into two major histopathologies, i.e., alveolar (ARMS) and embryonal (ERMS) (1, 2). Recent data obtained from two next-generation sequencing (NGS) studies revealed that RMS harbor a high rate of recurrent mutations in the RAS pathway (3, 4).Whole-genome and whole-exome sequencing of 147 tumor/normal pairs showed recurrent alterations in the RAS genes predominantly in the ERMS subtype, i.e., NRAS in 11.7%, KRAS in 6.4%, and HRAS in 4.3% of cases (4). In an independent study, genomic analysis of 13 primary RMS samples and matched normal tissue revealed that the most common cancer consensus gene mutations in RMS were in the RAS pathway, including mutations in NRAS, KRAS, and HRAS (3). In this study, 75% (6/8) of high-risk ERMS tumors harbored RAS pathway mutations and these mutations were significantly associated with risk-group assignment (3). Additional studies documented activation of the RAS pathway by oncogenic mutations in HRAS, KRAS, or NRAS in RMS, i.e., in 42% [12/26] of RMS (5) and in 35% (5/14), 22% (7/31) (6, 7), and 11.7% (8) of ERMS tumors.

RAS proteins constitute key components of cellular signaling pathways originating from cell surface receptors (9). Oncogenic RAS proteins control a complex molecular network including cell survival as well as cell death pathways (9). Also, oncogenic RAS has been implicated in regulating the sensitivity of cancer cells to oxidative stress (10). Depending on the cellular context, e.g., on the sensitivity toward apoptotic stimuli and the status of RAS effector pathways, oncogenic RAS proteins may exert antiapoptotic and proapoptotic functions (9).

Despite the documented relevance of oncogenic RAS to drive tumorigenesis of RMS, little is yet known about the impact on cell death and survival signaling pathways. In the present study, we therefore investigated the role of oncogenic RAS genes in the control of cell death of RMS.

## Materials and Methods

### Cell culture and chemicals

RMS13 cells were obtained from the American Type Culture Collection (Manassas, VA, USA) and maintained in RPMI 1640 medium (Life Technologies, Eggenstein, Germany), supplemented with 10% fetal calf serum (FCS) (Biochrom, Berlin, Germany), 1 mM glutamine, and 1% penicillin/streptomycin (Invitrogen, Karlsruhe, Germany). PI3K/mTOR inhibitor PI103 (11) was purchased from Merck Millipore (Darmstadt, Germany), RSL3 from InterBIOScreen Ltd. (Moscow, Russia), Auranofin from Santa Cruz Biotechnology Inc. (Santa Cruz, CA, USA). All chemicals were purchased from Sigma (Deisenhofen, Germany) unless indicated otherwise.

### Transduction

For overexpression of RAS mutants, RMS13 cells were transduced with *pMSCV-puro* vector containing oncogenic RAS mutants (i.e., NRAS12V, KRAS12V, or HRAS12V; respective vectors were sequenced to verify the identity of the individual mutant RAS) or empty vector using the packaging cell line Platinum-E. Stable cell lines were selected with puromycin.

### Determination of cell viability, cell density, cell count, colony formation, apoptosis, and cell death

Cell viability was assessed by 3-(4,5-dimethylthiazol-2-yl)-2,5-diphenyltetrazolium bromide (MTT) assay according to the manufacturer’s instructions (Roche Diagnostics, Mannheim, Germany). Cell density was assessed by crystal violet staining (0.75% crystal violet, 50% ethanol, 0.25% NaCl, 1.57% formaldehyde). Crystal violet dye was resolubilized in 1% sodium dodecyl sulfate (SDS) and absorbance at 550 nM was measured by microplate reader (Infinite M200, Tecan Group Ltd., Maennedorf, Switzerland). Cell counts were determined by CASY cell counter (OLS OMNI Life Science, Bremen, Germany). Apoptosis was determined by analysis of DNA fragmentation of propidium iodide (PI)-stained nuclei using flow cytometry (FACSCanto II, BD Biosciences, Heidelberg, Germany), as described previously (12). Cell death was assessed by measuring loss of plasma membrane integrity by PI-emitted fluorescence and flow cytometry. For colony assay, cells were seeded as single cells (200 cells/well) in six-well plates and cultured for 10 days before colonies were stained with crystal violet (Roth, Karlsruhe, Germany) and counted.

### Western blot analysis

Western blot analysis was performed as described previously (12) using the following antibodies: mouse anti-AKT (BD Biosciences), rabbit anti-pAKT, rabbit anti-p4E-BP1, rabbit anti-4E-BP1, rabbit anti-pS6, mouse anti-S6, rabbit anti-pERK, rabbit anti-ERK, rabbit anti-pan-RAS (Cell Signaling, Beverly, MA, USA). Mouse anti-GAPDH (HyTest, Turku, Finland) or mouse anti-β-Actin (Sigma) were used as loading controls. Goat anti-mouse IgG, donkey anti-goat IgG, goat anti-rabbit IgG conjugated to horseradish peroxidase (Santa Cruz Biotechnology Inc.), and goat anti-mouse IgG1 or goat anti-mouse IgG2b (Southern Biotech, Birmingham, AL, USA) conjugated to horseradish peroxidase were used as secondary antibodies. Enhanced chemiluminescence was used for detection (Amersham Bioscience, Freiburg, Germany). Also, donkey anti-mouse IgG or donkey anti-rabbit (LI-COR Biotechnology, Bad Homburg, Germany) labeled with IRDye infrared dyes were used for detection. Representative blots of at least two independent experiments are shown.

### Statistical analysis

Statistical significance was assessed by Student’s *t*-test (two-tailed distribution, two-sample, unequal variance).

## Results

### Effects of oncogenic *RAS* genes on RAS/MEK/ERK and PI3K/AKT/mTOR signaling of RMS13 cells

**Figure 1:**
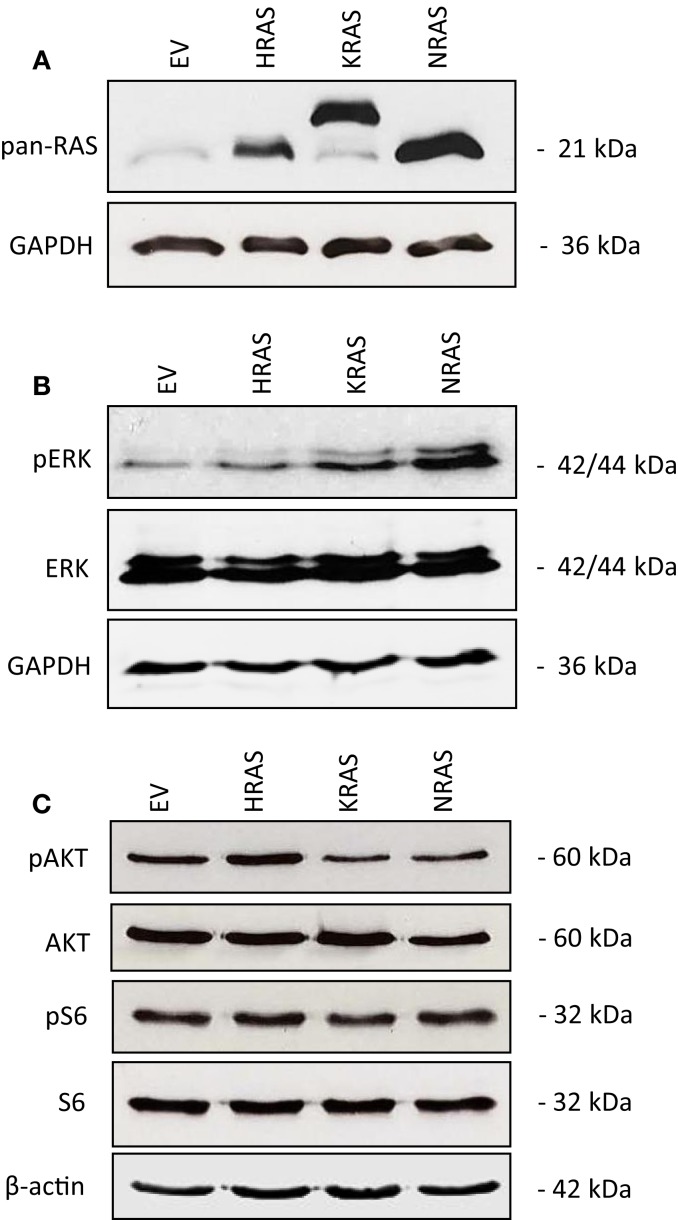
**Effects of oncogenic *RAS* genes on RAS/MEK/ERK and PI3K/AKT/mTOR signaling of RMS13 cells**. RMS13 cells expressing empty vector (EV), HRAS12V, KRAS12V, or NRAS12V were analyzed for RAS protein expression using a pan-RAS antibody **(A)**, for expression and phosphorylation of ERK **(B)**, and for expression and phosphorylation of AKT and S6 ribosomal protein **(C)** by Western blotting. Representative blots are shown.

To investigate the impact of oncogenic mutant variants of RAS in RMS, we ectopically expressed *NRAS12V*, *KRAS12V*, or *HRAS12V* in the RMS cell line RMS13 that harbors wild-type RAS. Ectopic expression of mutant *RAS* genes was confirmed by Western blot analysis using a pan-RAS antibody (Figure 1A). To determine whether overexpression of mutant *RAS* genes affects activation of RAS/MEK/ERK and/or PI3K/AKT/mTOR pathways, we assessed in parallel the phosphorylation status of key components of these pathways. Overexpression of mutant *RAS* genes resulted in increased phosphorylation of ERK or AKT (Figures 1B,C; Figure S1 in Supplementary Material), indicating that overexpression of mutant *RAS* genes results in increased activation of downstream signaling pathways.

### Effects of oncogenic RAS genes on cell numbers and clonogenic growth of RMS13 cells

**Figure 2:**
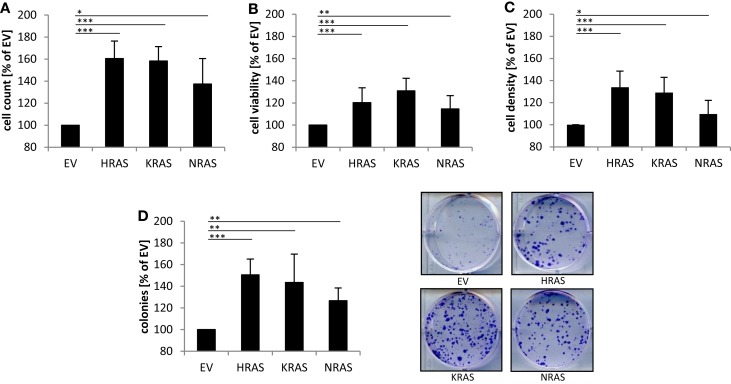
**Effects of oncogenic *RAS* genes on cell numbers and clonogenic growth of RMS13 cells**. RMS13 cells expressing EV, HRAS12V, KRAS12V, or NRAS12V were incubated for 48 h and analyzed for cell counts **(A)**, cell viability using MTT assay **(B)**, and cell density using crystal violet assay **(C)**; results are expressed as percentage of cells expressing EV. Clonogenic survival was assessed by colony formation assay at day 10 **(D)**. The number of colonies was counted after crystal violet staining and is expressed as percentage of cells expressing EV [**(D)**, left panel]; representative images are shown [**(D)**, right panel]. Mean + SD of three independent experiments performed in triplicate are shown; *p < 0.05; **p < 0.01; ***p < 0.001.

Next, we investigated the effects of mutant *RAS* genes on cell numbers. Ectopic expression of *NRAS12V*, *KRAS12V*, and *HRAS12V* all caused a significant increase in cell numbers compared to cells expressing empty control vector (Figure 2A). In addition, overexpression of mutant *RAS* genes significantly increased cell viability as determined by MTT assay (Figure 2B). Besides MTT assay, which relies on mitochondrial activity and may not reliably assess cell viability under oxidative stress, we also used crystal violet assay as another assay to determine cell viability, which yielded similar results (Figure 2C). In addition to these short-term assays, we also assessed long-term effects using colony assays to determine clonogenic survival. Of note, ectopic expression of NRAS12V, KRAS12V, and HRAS12V resulted in a significant increase in colony numbers compared to cells transduced with empty control vector (Figure 2D). This set of experiments shows that overexpression of mutant RAS genes increases cell numbers and clonogenic survival of RMS13 cells.

### Effects of oncogenic RAS genes on spontaneous cell death of RMS13 cells

**Figure 3:**
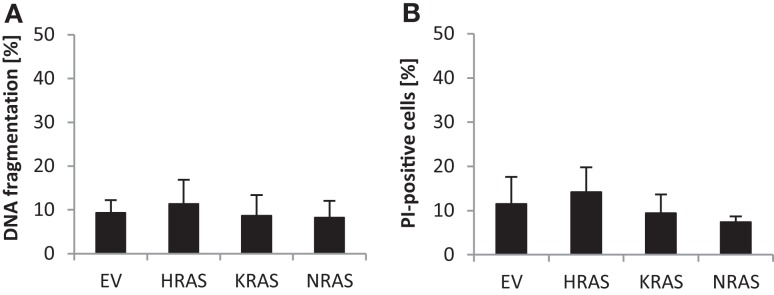
**Effects of oncogenic *RAS* genes on spontaneous cell death of RMS13 cells**. RMS13 cells expressing EV, HRAS12V, KRAS12V, or NRAS12V were incubated for 48 h. Apoptosis was determined by analysis of DNA fragmentation of PI-stained nuclei **(A)**, and cell death was determined by PI staining **(B)** using flow cytometry. Mean + SD of three independent experiments performed in triplicate are shown.

Since RAS has been implicated in the regulation of cell death in addition to cell growth, we also determined spontaneous cell death of untreated RMS13 cells in the absence of any cytotoxic stimulus. Analysis of DNA fragmentation, used as a characteristic marker of apoptotic cell death, showed no significant changes in DNA fragmentation upon overexpression of NRAS12V, KRAS12V, or HRAS12V compared to cells expressing empty control vector (Figure 3A). Similarly, overexpression of mutant RAS genes did not result in enhanced plasma membrane permeability as assessed by PI staining that was used as a marker of non-apoptotic cell death (Figure 3B). Based on these results, we conclude that ectopic expression of NRAS12V, KRAS12V, and HRAS12V does not increase spontaneous cell death of RMS13 cells.

### Oncogenic RAS genes rescue dual PI3K/mTOR inhibitor-mediated cytotoxicity

**Figure 4:**
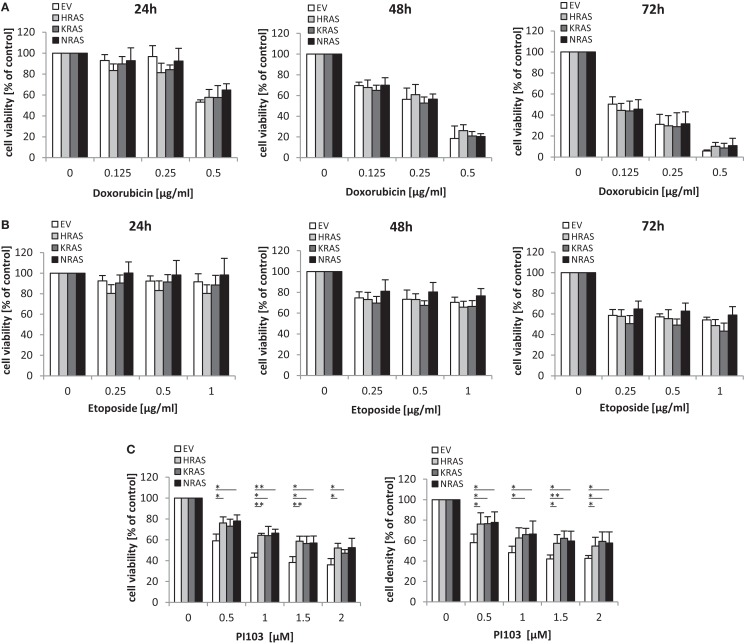
**Oncogenic *RAS* genes rescue dual PI3K/mTOR inhibitor-mediated cytotoxicity**. RMS13 cells expressing EV, HRAS12V, KRAS12V, or NRAS12V were treated for 24–72 h **(A,B)** or 48 h **(C)** with indicated concentrations of Doxorubicin **(A)**, Etoposide **(B)**, or PI103 **(C)**. Cell viability was determined by MTT assay and cell density by crystal violet assay; results are expressed as percentage of untreated cells. Mean + SD of three independent experiments performed in triplicate are shown; *p < 0.05; **p < 0.01.

Next, we investigated the question whether oncogenic RAS mutants affect the sensitivity of RMS13 cells toward anticancer agents. To this end, we tested the cytotoxicity of Doxorubicin or Etoposide, two chemotherapeutic drugs that are commonly used in clinical protocols for the treatment of RMS. Dose response and kinetic analysis showed that Doxorubicin and Etoposide reduced cell viability of RMS13 cells in a concentration- and time-dependent manner irrespective of whether or not NRAS12V, KRAS12V, or HRAS12V were ectopically expressed (Figures 4A,B). By contrast, overexpression of NRAS12V, KRAS12V, or HRAS12V significantly rescued loss of cell viability or cell density upon treatment with the dual PI3K/mTOR inhibitor PI103 (Figure 4C).

### Oncogenic *RAS* genes protect against oxidative stress stimuli

**Figure 5:**
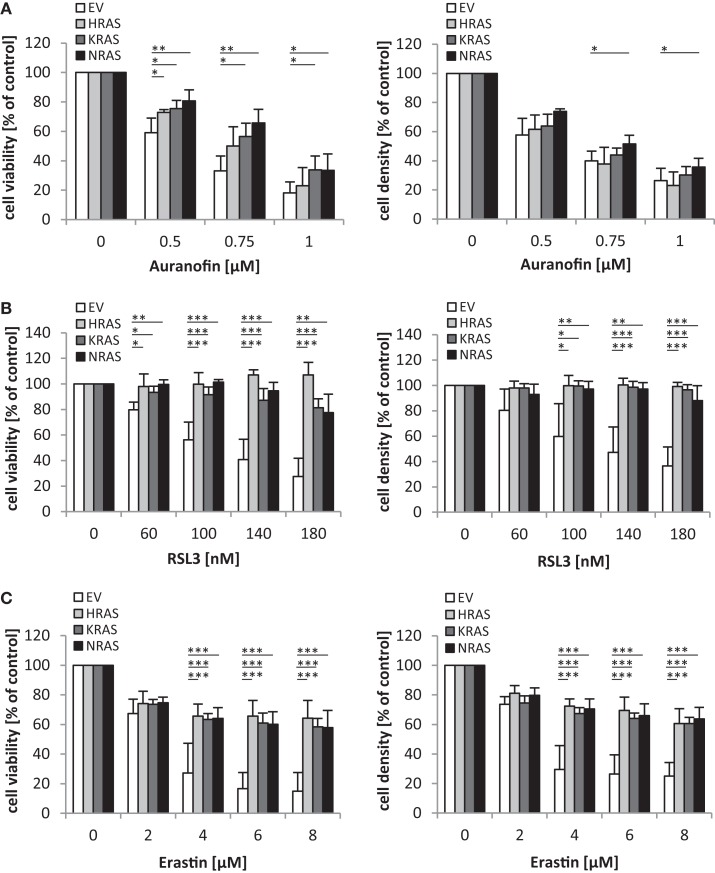
**Oncogenic *RAS* genes protect against oxidative stress stimuli**. RMS13 cells expressing EV, HRAS12V, KRAS12V, or NRAS12V were treated for 48 h with indicated concentrations of Auranofin **(A)**, RSL3 **(B)**, or Erastin **(C)**. Cell viability was determined by MTT assay and cell density by crystal violet assay; results are expressed as percentage of untreated cells. Mean + SD of three independent experiments performed in triplicate are shown; *p < 0.05; **p < 0.01; ***p < 0.001.

Since oncogenic RAS has been implicated in regulating the sensitivity of cancer cells to oxidative stress (10), we extended our study to several agents that interfere with antioxidative defense mechanisms and thereby increase reactive oxygen species (ROS) levels. Interestingly, we found that overexpression of NRAS12V, KRAS12V, or HRAS12V significantly protected RMS13 cells against loss of cell viability and reduction of cell density upon treatment with Auranofin (Figure 5A), an inhibitor of thioredoxin reductase (13). Also, RMS13 cells engineered to overexpress NRAS12V, KRAS12V, or HRAS12V were significantly more resistant to RSL3 (Figure 5B), a pharmacological inhibitor of glutathione (GSH) peroxidase 4 (GPX4) (14). GPX4 is the only GPX that specifically reduces hydroperoxides within membranes (15). In addition, RMS13 cells exhibiting oncogenic RAS mutants were significantly less susceptible against Erastin (Figure 5C). Erastin is an inhibitor of system ${x_{c}}^{-}$, a cysteine/glutamate amino acid transporter at the plasma membrane (10), and inhibits GSH synthesis by blocking cysteine uptake. Together, this set of experiments demonstrates that oncogenic RAS mutants protect RMS13 cells against several oxidative stress stimuli.

### Oncogenic *RAS* genes protect against ferroptotic cell death

**Figure 6:**
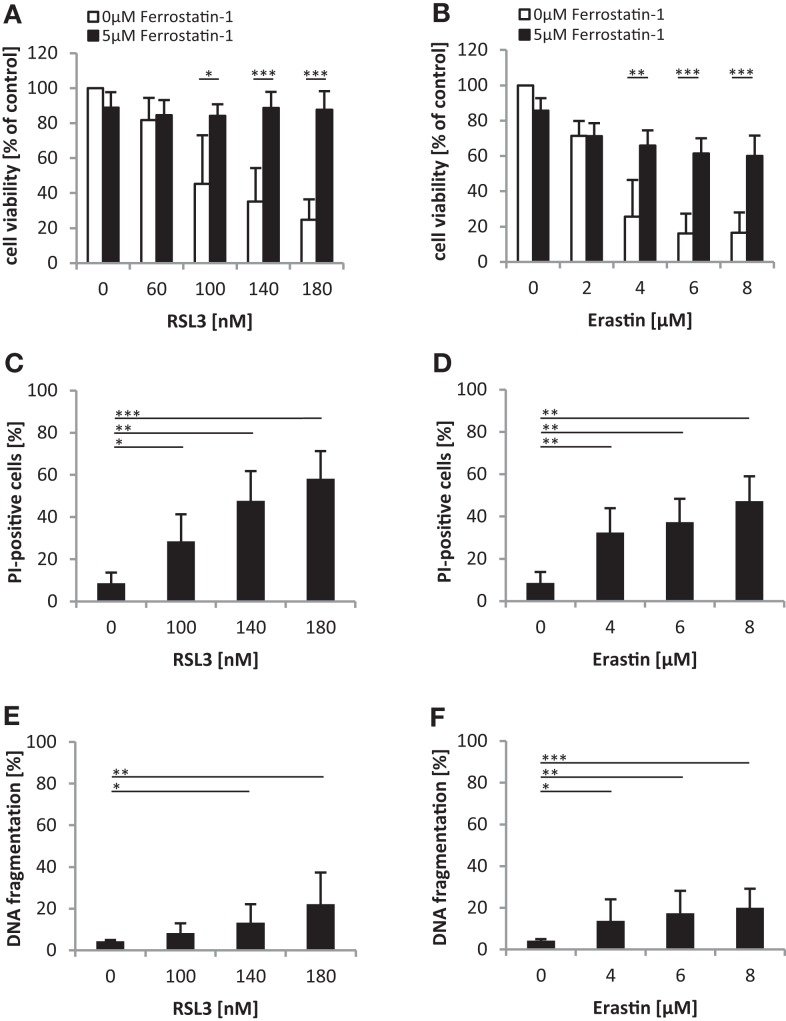
**Oncogenic *RAS* genes protect against ferroptotic cell death**. **(A,B)** RMS13 cells expressing EV were treated for 48 h with indicated concentrations of RSL3 **(A)** or Erastin **(B)** in the presence or absence of 5 μM Ferrostatin-1. Cell viability was determined by MTT assay; results are expressed as percentage of untreated cells. Mean + SD of three independent experiments performed in triplicate are shown; *p < 0.05; **p < 0.01; ***p < 0.001. **(C–F)** RMS13 cells expressing EV were treated for 48 h with indicated concentrations of RSL3 or Erastin. Apoptosis was determined by analysis of DNA fragmentation of PI-stained nuclei **(C,D)** and cell death was determined by PI staining **(E,F)** using flow cytometry. Mean + SD of three independent experiments performed in triplicate are shown; *p < 0.05; **p < 0.01; ***p < 0.001.

We noted that oncogenic RAS mutants conferred protection against both RSL3 and Erastin, which either directly (i.e., RSL3) or indirectly through GSH depletion (i.e., Erastin) inhibit GPX4 (14). Since GPX4 has recently been identified as an essential regulator of ferroptosis (14), an iron-dependent non-apoptotic mode of cell death (16), we asked whether RSL3 and Erastin trigger ferroptotic cell death in RMS13 cells. To address this question, we used Ferrostatin-1, which has been described to block ferroptosis (10). Indeed, addition of Ferrostatin-1 significantly reduced RSL3- or Erastin-induced loss of cell viability (Figures 6A,B). To further test whether RSL3 and Erastin engage a non-apoptotic form of cell death, we assessed in parallel plasma membrane permeability using PI-staining and DNA fragmentation as markers of non-apoptotic and apoptotic cell death, respectively. Notably, treatment with RSL3 or Erastin caused a significant increase in plasma membrane permeability as reflected by increased PI positivity (Figures 6C,D), whereas only a minor increase in the rate of DNA fragmentation was observed (Figures 6E,F), consistent with a non-apoptotic mode of cell death. Together, this set of experiments indicates that RSL3 and Erastin trigger ferroptotic cell death in RMS13 cells.

## Discussion

In the present study, we investigated the role of oncogenic RAS genes in the regulation of cell death of RMS13 cells. A key finding of our study is the increased resistance to oxidative stress that is conferred by ectopic expression of oncogenic RAS mutants. RMS13 cells engineered to express NRAS12V, KRAS12V, or HRAS12V proved to be significantly less vulnerable to several redox-targeting agents that inhibit antioxidative defense systems responsible for ROS detoxification. This increased resistance to oxidative stress occurs upon inhibition of distinct antioxidative defense pathways, including the GSH system (that is inhibited by Erastin and RSL3) as well as the thioredoxin system (that is inhibited by Auranofin), emphasizing the general relevance of this finding. Interestingly, this form of oxidative stress-induced cell death turned out to be ferroptosis, a recently defined iron-dependent form of programed cell death involving ROS production (16). Our rescue experiments showing that Ferrostatin-1 confers protection against Erastin- or RSL3-induced cytotoxicity underscores that these compounds trigger ferroptotic cell death in RMS13 cells that is attenuated by oncogenic RAS mutants.

Of note, our key finding showing that the RAS mutation status imparts resistance toward treatment with ferroptosis-inducing compounds such as Erastin and RSL3 is in line with recent evidence documenting that the RAS mutation status does not predict sensitivity to Erastin (14). A large analysis of more than a hundred of different cancer cell lines recently documented no selective lethality of Erastin in RAS-mutated cancer cell lines over RAS wild-type counterparts (14). This comprehensive study indicates that oncogenic RAS does not confer sensitivity to Erastin across cancers. By comparison, Erastin has been reported to exhibit greater lethality in human tumor cells harboring mutations in the oncogenes *HRAS*, *KRAS*, or *BRAF* (17) as well as in an individual genetic context using isogenic cell lines with and without oncogenic *RAS* genes (10). Thus, there are likely to be found other more dominant determinants of sensitivity toward Erastin than RAS mutations when analyzing sensitivity across diverse contexts.

In addition to redox-targeting agents, oncogenic RAS mutants also conferred resistance to the dual PI3K/mTOR inhibitor PI103, while they did not alter the response to DNA-damaging chemotherapeutics such as Etoposide and Doxorubicin. This suggests that oncogenic RAS selectively modulates cell death pathways in response to cytotoxic stimuli in RMS13 cells.

Oncogenic forms of RAS have previously been implicated in the control of both proliferation and cell death of cancer cells (9). Consistent with the well-documented role of oncogenic RAS to drive cell cycle progression and clonogenic growth, RMS13 cells harboring NRAS12V, KRAS12V, or HRAS12V exhibited a significant increase in proliferation and colony formation as compared to cells with wild-type RAS. While oncogenic RAS has been described to also promote cell death under certain circumstances (9), we found no evidence of increased spontaneous cell death in the absence of lethal insults in RMS13 cells, neither apoptotic nor non-apoptotic cell death.

Several genomic studies of RMS samples have revealed a high rate of recurrent mutations in the RAS pathway, which is associated with intermediate and high-risk disease (3). This underscores that RAS signaling is a clinically relevant oncogenic pathway in RMS. Our present study contributes to a better understanding of the biology of oncogenic RAS in RMS. While RAS remains one of the most elusive genes to target directly, RAS mutant cells have been shown to depend on a number of oncogenic signaling pathways that arise as a means of adaptation to RAS-driven intracellular stresses and represent unique vulnerabilities of mutant RAS cancers (18). In RMS, concomitant inhibition of the RAS/MEK/ERK and PI3K/AKT/mTOR pathways has recently been demonstrated in two independent studies to synergistically trigger apoptosis and to inhibit tumor growth *in vivo* (19, 20). Thus, therapeutic targeting of RAS effector pathways and the search for synthetic lethal interactors of mutant RAS may offer exiting opportunities for new therapeutic directions.

## Author Contributions

CS performed experiments, analyzed and interpreted data; NC performed experiments; UG analyzed and interpreted data; HH designed research, analyzed and interpreted data; SF designed research, analyzed and interpreted data, and wrote the manuscript; all authors approved the final version of the paper.

## Conflict of Interest Statement

The authors declare that the research was conducted in the absence of any commercial or financial relationships that could be construed as a potential conflict of interest.

## Supplementary Material

The Supplementary Material for this article can be found online at http://journal.frontiersin.org/article/10.3389/fonc.2015.00131
