# Supplementary figures and images for: Oncogenic RAS Mutants Confer Resistance of RMS13 Rhabdomyosarcoma Cells to Oxidative Stress-Induced Ferroptotic Cell Death
Source: Front Oncol. 2015 Jun 22;5:131. doi: 10.3389/fonc.2015.00131 (PMC4476278; doi:10.3389/fonc.2015.00131)

Suppl. Figure 1

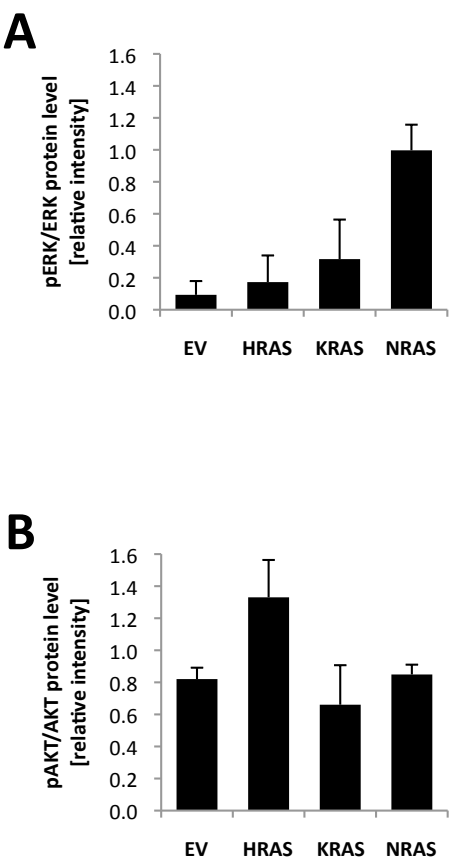

Supplement: Supplementary file 1 [file image_1.pdf]
